# The Alzheimer's Disease-Associated R47H Variant of TREM2 Has an Altered Glycosylation Pattern and Protein Stability

**DOI:** 10.3389/fnins.2016.00618

**Published:** 2017-01-18

**Authors:** Ji-Seon Park, In Jung Ji, Dong-Hou Kim, Hyun Joo An, Seung-Yong Yoon

**Affiliations:** ^1^Alzheimer's Disease Experts Lab, Asan Medical Center, University of Ulsan College of MedicineSeoul, South Korea; ^2^Department of Brain Science, University of Ulsan College of MedicineSeoul, South Korea; ^3^Bio-Medical Institute of Technology, University of Ulsan College of MedicineSeoul, South Korea; ^4^Cell Dysfunction Research Center, University of Ulsan College of MedicineSeoul, South Korea; ^5^Asia Glycomics Reference SiteDaejeon, South Korea; ^6^Graduate School of Analytical Science and Technology, Chungnam National UniversityDaejeon, South Korea

**Keywords:** Alzheimer's disease, glycosylation, Nasu-Hakola disease, TREM2, trafficking

## Abstract

The R47H coding variant of the triggering receptor expressed on myeloid cells-2 (TREM2) increases the risk of Alzheimer's disease (AD) similar to apolipoprotein E4. TREM2 R47H has recently been shown to have impaired binding to damage-associated lipid or apolipoprotein ligands. However, it is not known how this R47H variant affects the biochemical characteristics of TREM2 and alters the pathogenesis of AD. We previously reported that TREM2-R47H has a slightly different glycosylation pattern from wild-type. A more detailed characterization in our present study confirms that TREM2 R47H has an altered glycosylation pattern and reduced stability. TREM2 R47H shows different glycosylation profiles from analysis using monensin or kifunensine treatment which were confirmed by mass spectrometry. The solubility of TREM2 R47H and its cleaved products such as intracellular domain (ICD) is also decreased, increasing its proteasomal and lysosomal degradation. The different biochemical characteristics of TREM2 R47H, including glycosylation, solubility and processing, may offer insights into a future therapeutic strategy for AD.

## Introduction

**Figure 1:**
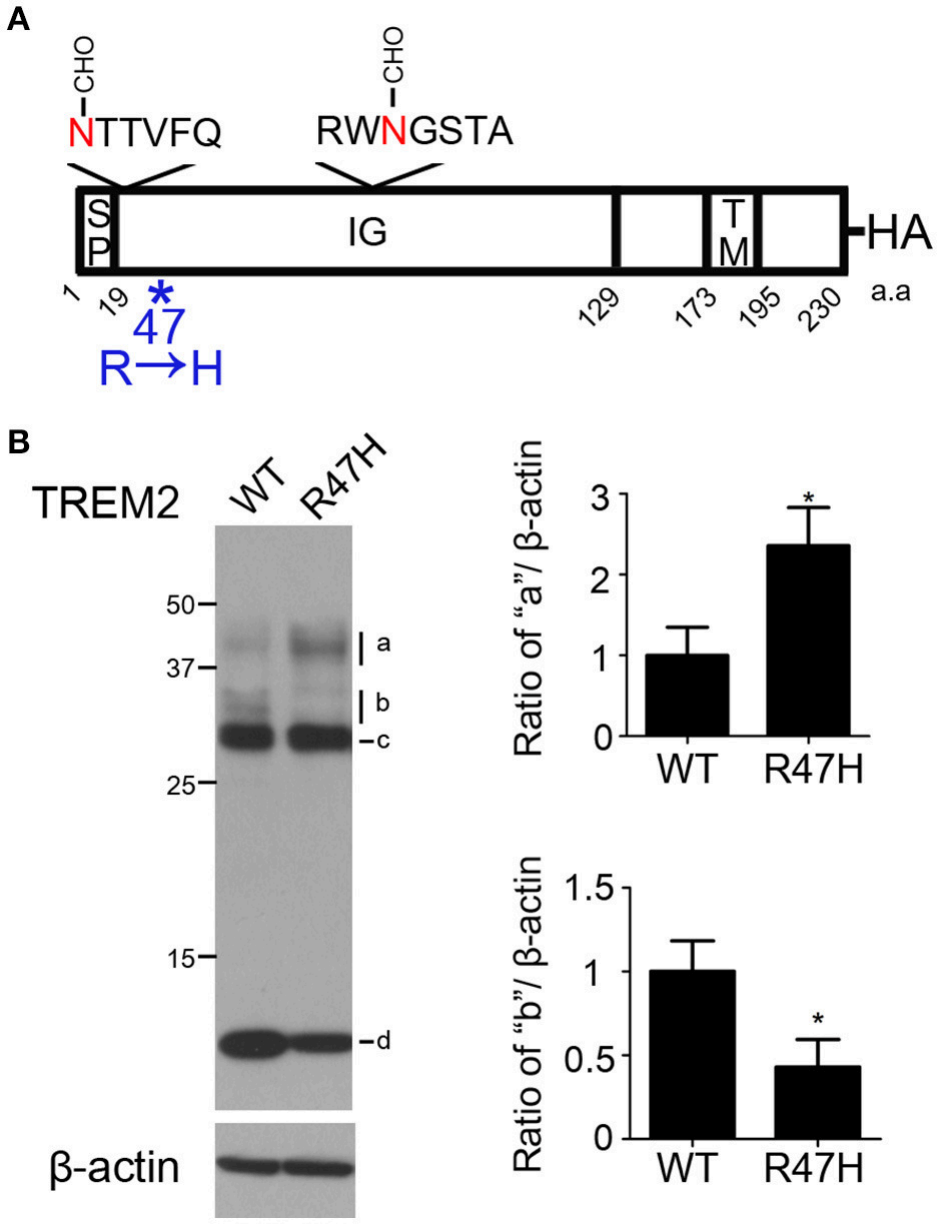
**Differences in the wild-type and R47H variant TREM2 expression patterns. (A)** Schematic diagram of the triggering receptor expressed on myeloid cells-2 (TREM2) showing 2 N-glycosylation sites on the immunoglobulin (IG) domain of TREM2 and mutation site R47H. The hemagglutinin (HA)-tag was added to the C-terminus. SP, signal peptide. TM, transmembrane domain. **(B)** HeLa cells were transfected with wild-type TREM2 and its R47H mutant and immunoblotted with HA antibody. A similar thick band of ~28 kDa was found for both expressed proteins (c) with different patterns found for the upper bands (a,b) and thick lower bands (d). These data represent the mean ± *S.E*. from three independent experiments. ^*^P < 0.05.

Myeloid cells such as microglia, dendritic cells, osteoclasts, and macrophages express triggering receptor expressed on myeloid cells-2 (TREM2) which belongs to the immunoglobulin superfamily. TREM2 is a type 1 membrane protein consisted of an extracellular immunoglobulin-like domain and a short cytoplasmic tail (Figure 1A). TREM2 may work in myeloid cells, most presumably osteoclasts and microglial cells, both of which are included in brain function and bone modeling (Cella et al., 2003). Microglial TREM2 also has an important role in removing neural debris in the brain lesion (Lucin et al., 2013).

The R47H variant of TREM2 was recently revealed to be associated with the progression of late-onset Alzheimer's disease (AD) by a genome-wide-association study (GWAS, Jiang et al., 2013). TREM2 is increased in the microglia in AD transgenic mouse models (Frank et al., 2008; Melchior et al., 2010). A fronto-temporal dementia (FTD)-like syndrome without bone pathology was also recently reported to be associated with several TREM2 mutations (Guerreiro et al., 2013). TREM2 mutations have also been identified as risk factors for Parkinson's disease (PD), amyotrophic lateral sclerosis (ALS), and FTD (Rayaprolu et al., 2013; Borroni et al., 2014; Cady et al., 2014; Cuyvers et al., 2014). TREM2 mutations such as Y38C and T66M are related to a genetic disease known as Nasu-Hakola disease [NHD, polycystic lipomembranous osteodysplasia with sclerosing leukoencephalopathy (PLOSL)] (Soragna et al., 2003). NHD is an autosomal, recessive disorder which occurs in families from Finland and Japan. It shows progressive, presenile, inflammatory neurodegeneration, and the formation of bone cysts. Severe brain lesions were also previously identified in mice with the same mutations (Colonna, 2003; Kaifu et al., 2003).

Several different ligands for TREM2 have been reported including apolipoprotein E (Atagi et al., 2015; Bailey et al., 2015), lipids exposed upon axonal injury (Poliani et al., 2015), nucleic acids released from dying cells (Kawabori et al., 2015) and damage-associated molecular signatures found on bacteria (Daws et al., 2003; N'Diaye et al., 2009). Interestingly, the AD-associated TREM2 R47H variant has impaired binding to the apolipoprotein E or injury-associated lipids (Atagi et al., 2015; Bailey et al., 2015; Poliani et al., 2015). However, it is still unknown how this impairment occurs, or how this may relate to AD pathogenesis. Hence, although it is now known that the glycosylation and trafficking of TREM2 is impaired by NHD-associated mutations such as Y38C and T66M (Kleinberger et al., 2014; Park et al., 2015), it is not yet clear how the biochemical characteristics of TREM2 are affected by the AD-associated R47H variant. Although we previously reported that TREM2-R47H shows subtle glycosylation pattern changes (Park et al., 2015), a more detailed characterization of this variant is necessary to provide further insights into the molecular basis for AD pathogenesis. Hence, we here compared the biochemical characteristics of TREM2 R47H and wild-type TREM2 in more detail to further elucidate the disruption to TREM ligand binding and promotion of AD pathogenesis by this variation.

## Methods

### Reagents

MG132 was obtained from Calbiochem (San Fransico, CA, USA). Monensin, kifunensin, cycloheximide, bafilomycin A1 were sourced from Sigma-Aldrich (St Louis, MO). All plasmids used for cloning were obtained from Cosmo Genetech Co. Ltd.

### Plasmid constructions and antibodies

Human TREM2 construct was purchased from Sino Biological Inc., BDA, Beijing and pcDNA5-FRT/TO-HA was obtained from Dr. SW Kang Bio-Medical Institute of Technology. Wild-type human TREM2 (TREM2 wild-type) was inserted into Hind-III and Xho-I sites of pcDNA5-FRT/TO-HA. A point mutation in TREM2 was generated to substitute Arg with His at position 47 and thus produce the R47H variant. These modified TREM2 mutations of cDNAs were PCR-amplified and sub-cloned into the pcDNA5-FRT/TO-HA vector. All constructs was verified by DNA sequencing.

### Cell-culture analysis

HeLa cells were maintained in DMEM (HyClone Laboratories, UT, USA) supplemented with 10% fetal bovine serum (HyClone Laboratories) and were incubated in 5% CO_2_ at 37°C. All experiments involving transient transfection were performed using Lipofectamine 2000 (Invitrogen, Carlsbad, CA) in accordance with the manufacturer's instructions. Cells transfected with the wild-type and R47H variant of TREM2 were incubated in antibiotic-free DMEM media for 4 h, after which the media was replaced with DMEM supplemented with FBS.

### Protein extraction and analysis

Cell lysates were prepared in a buffer containing 1% SDS, 100 mM Tris-HCl, and pH 8.0 and were boiled for 5 min. To obtain Triton X-100-soluble and -insoluble fractions for the analysis of protein solubility, cells were incubated with Triton-lysis-buffer [1% Triton X-100, 20 mM HEPES pH7.5, 150 mM NaCl, protease inhibitor cocktail (Calbiochem)] for 15 min on a rocking platform as previously described (Kang et al., 2006). After centrifugation, the supernatant (Triton-soluble fraction) was collected. The Triton insoluble fraction (pellet) was resuspended in SDS-lysis-buffer (1% SDS, 100 mM Tris-HCl, and pH 8.0). Both fractions were reconstituted to equal volumes and analyzed by western blotting as described below.

To analyze the digitonin fraction, cells were incubated with 0.75% digitonin solution for 5 min on ice as previously described (Kang et al., 2006). Parts of the lysate were mixed with the sample buffer for the preparation of total cell sample and some of lysate was centrifuged at 800 g for 5 min. Supernatant (cytosol fraction) was collected and pellet were incubated with Triton-lysis-buffer (1% Triton X-100, 20 mM HEPES pH 7.5, 150 mM NaCl, protease inhibitor cocktail) for 5 min on ice. The pellet incubated with Triton-lysis buffer were centrifuged at 800 g for 5 min. Some of supernatant was collected for the membrane fraction and the remainder was centrifuged at 13,000 rpm for 30 min (the supernatant contained the soluble membrane fraction and the pellet the insoluble membrane fraction).

### Western blotting

For western blotting, protein lysates from HeLa cells transfected with either the TREM2 wild-type or R47H mutant construct were fully solubilized with 1% SDS, and 100 mM Tris-HCl, pH 8.0. Protein concentrations were measured using the Bradford method. Equal amounts of protein were mixed with the sample buffer (62.5 mM Tris-HCl [pH 6.8], 1% [w/v] sodium dodecyl sulfate [SDS], 2.5% [v/v] glycerol, 0.5% [v/v] β-mercaptoethanol, and bromophenol blue), boiled at 100°C for 5 min, and stored at −20°C until use. Proteins were resolved by SDS-polyacrylamide gel electrophoresis at a constant voltage (100 V) and were subsequently transferred to polyvinylidene difluoride membranes (pore size, 0.2 mm; BioRad, CA) at 100 V for 1.5 h. After 1 h incubation in blocking PBST buffer (0.1% [v/v] Tween-20) containing 2% (w/v) bovine serum albumin and 2% (v/v) normal horse serum, blots were incubated with primary antibodies overnight at 4°C. Blots were then washed in PBST buffer, incubated with horseradish, peroxidase-conjugated, anti-IgG (1:5000; Pierce, Rockford, IL), and visualized using enhanced chemiluminescence reagents (Amersham, Arlington Heights, IL) and X-ray film. The primary antibodies used in the immunoblotting were rat anti-HA (1:5000; Roche, Mannheim, Germany), mouse anti-GRP78 (1:1000, BD Transduction Laboratories™, California, CA, USA), rabbit anti-phospho-eIF2α (Ser51) (1:1000, Cell Signaling Technology, Danvers, MA), rabbit anti-phospho PERK(Thr981) (1:100, Santa Cruz, CA), rabbit anti-PERK(H-300) (1:100, Santa Cruz, CA), and mouse anti-β-actin (1:10,000; Sigma-Aldrich).

### In-gel PNGase F digestion

Coomassie blue-stained protein bands were excised from the ge1, chopped into 1 mm^3^ pieces and washed repeatedly in 100 mM ammonium bicarbonate buffer for 10 min and then in acetonitrile for 10 min to enable enzyme penetration. The pieces were then dried thoroughly in a vacuum centrifuge and rehydrated in 200 mM ammonium bicarbonate containing 1 μL (or 500 U) of peptide N-glycosidase F (New England Biolabs). Enzymatic deglycosylation was then performed at 37°C for 16 h in the water bath. At the end of the digestion, the N-glycan supernatant was drawn out from the gel pieces and the extracted glycans were dried in a vacuum centrifuge.

### Nano-LC/MS analysis and data processing

MS analysis was performed in accordance with the optimized procedures published by Hua et al. (2013). Aqueous glycan solutions were injected by autosampler onto a chip-mounted nano-LC column (Agilent Technologies), consisting of a 9 × 0.075 mm i.d. enrichment column and a 43 × 0.075 mm i.d. analytical column. Both columns were packed with 5 μm porous graphitized carbon as the stationary phase. A rapid glycan elution gradient was delivered to the analytical column at 0.3 μL/min using solutions of (A) 3.0% acetonitrile and 0.1% formic acid (v/v) in water, and (B) 90.0% acetonitrile and 0.1% formic acid (v/v) in water, ramping from 0 to 44% B solution over 30 min. Remaining non-glycan compounds were flushed out with 100% B solution prior to re-equilibration. MS spectra were acquired via a TOF MS detector (model 6530, Agilent Technologies) in positive ionization mode over a mass range of m/z 500−2000 with an acquisition time of 1.5 s per spectrum. Following data acquisition, raw LC/MS data were processed using the Molecular Feature Extractor algorithm included in the MassHunter Qualitative Analysis software (Version B.6.00 SP1, Agilent Technologies). Using expected isotopic distribution and charge state information, extracted ion chromatograms were combined to create extracted compound chromatograms (ECCs) representing the summed signal from all ion species associated with a single compound (e.g., the doubly protonated ion, the triply protonated ion, and all associated isotopologues). Thus, each individual ECC peak could be taken to represent the total ion count associated with a single distinct compound. Each ECC peak was matched by accurate mass to a comprehensive library of all possible complex, hybrid, and high mannose glycan compositions based on known biosynthetic pathways and glycosylation patterns (Kronewitter et al., 2009). Deconvoluted masses of each ECC peak were compared against theoretical glycan masses using a mass error tolerance of 10 ppm. As samples originated from human cell lines, only glycan compositions containing hexose (Hex), N-acetylhexosamine (HexNAc), fucose (Fuc), and N-acetylneuraminic acid (NeuAc) were considered.

### Statistical analysis

Western bands were measured and analyzed with Image J software. Data are presented as mean ± SEM from at least triplicate experiments. Statistical analyses were performed using GraphPad Prism 5.0 software using an unpaired *t*-test or two-way ANOVA; ^***^P < 0.001; ^**^P < 0.01; ^*^P < 0.05.

## Results

### Differences in the wild-type and R47H variant TREM2 expression patterns

To investigate whether, and how, the R47H variant of TREM2 reported in AD (Soragna et al., 2003; Jiang et al., 2013) affected the trafficking of TREM2, the R47H mutation was introduced into TREM2 and the variant protein was expressed in HeLa cells (Figure 1B and Supplementary Figure 1). Western blots confirmed that the expression patterns of wild-type TREM2 and R47H mutants differed as we previously observed (Park et al., 2015). The expected molecular weight of TREM2 is about 25 kDa. Both the wild-type and R47H variant of TREM2 showed a thick band of ~28 kDa (Figure 1B, c) with the N-glycosylated form detectable in the ER. The upper 30~40 kDa bands denote the glycosylated forms in the Golgi. The uppermost bands of R47H at about 40 kDa were more intense than those of the wild-type protein (Figure 1B, a), although the upper bands of R47H at about 30 kDa were at a lower intensity than wild-type (Figure 1B, b). the C-terminal fragment (CTF) of R47H at about 10 kDa was less intense than its wild-type counterpart (Figure 1B, d).

### Increased terminal glycosylation of the TREM2 R47H variant in the trans-Golgi

**Figure 2:**
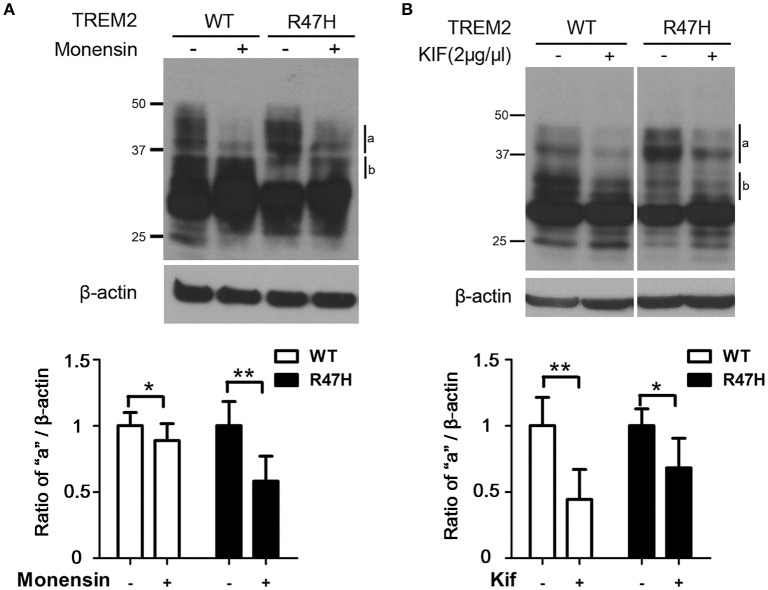
**Increased terminal glycosylation of the TREM2 R47H variant in the trans-Golgi. (A)** After monensin (10 μM) treatment for 8 h, the highest bands (a) for wild type TREM2 and the R47H variant were decreased in intensity, whereas the middle bands (b) were increased. **(B)** After kifunensin (2 μg/μl) treatment for 4 h, the intensity of the highest bands (a) for wild type TREM2 and the R47H variant were decreased. These data represent the mean ± *S.E*. from three independent experiments. ^*^P < 0.05, ^**^P < 0.01.

Since the upper bands for TREM2 on an immunoblot reflect its glycosylation status (Park et al., 2015), we suspected that the different expression patterns of wild-type TREM2 and its R47H variant (Figure 1B) are due to glycosylation changes. Hence, we treated cells with monensin (10 μM, 8 h) which blocks the transport from the medial to the trans cisternae of the Golgi stack and prevents terminal glycosylation (Griffiths et al., 1983). The highest western band (Figure 2A, a), which is more prominent for TREM2 R47H, is a monensin-sensitive glycosylated form but the lower band (Figure 2A, b), which is more prominent for the wild type protein, is resistant to monensin. Thus, the mobility difference between wild-type and R47H variant of TREM2 reflects the differences in their glycan structures.

To further analyze the glycosylation characteristics of TREM2, we treated cells with kifunensin, a mannosidase I inhibitor that prevents the trimming of mannose residues from high mannose N-glycans, thereby preventing the synthesis of complex N-glycans that are acceptors for α1–3-fucosyltransferases (Elbein et al., 1990; Yago et al., 2003). Kifunensine treatment decreased the intensity of the upper bands (Figure 2B, a and b), indicating that the higher TREM2 bands of ~30–45 kDa (Figure 2B, a and b) represent the addition of complex oligosaccharide chains to this protein in the Golgi and the thick, main band at about 28 kDa (Figure 1B, c) represents N-linked glycosylation with high mannose in the ER, further confirming our previous findings (Park et al., 2015).

### Different glycosylation profiles of wild-type TREM2 and the R47H variant

**Figure 3:**
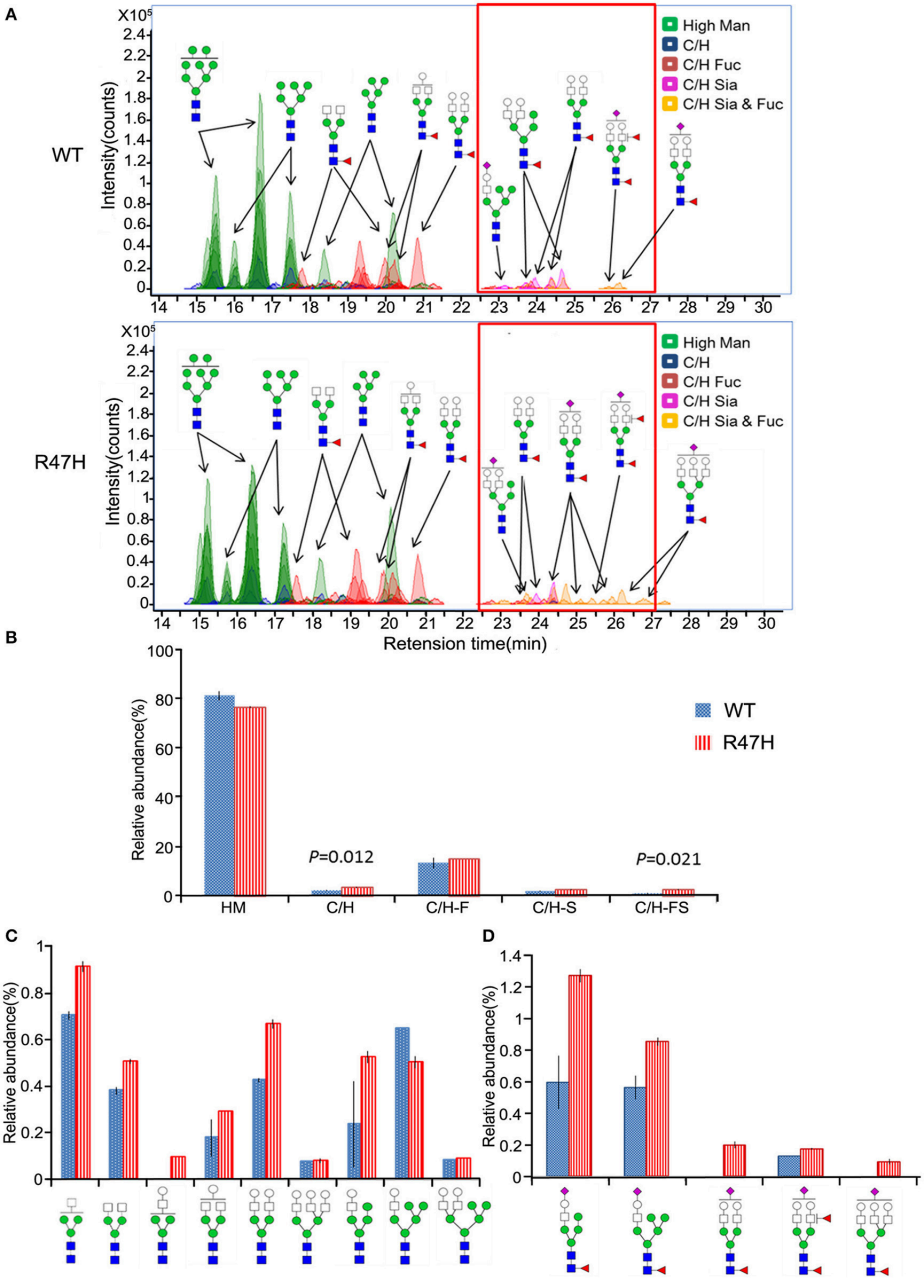
**Different glycosylation profiles of wild-type TREM2 and the R47H variant. (A)** Extracted compound chromatograms (ECCs) of N-glycans found in wild-type TREM2 (top) and the R47H variant (bottom). Colors denote different glycan classes. **(B)** Bar graph of relative abundances and standard errors associated with complex, hybrid, and high mannose type N-glycans (to indicate the statistically significant differences between wild-type TREM2 and the R47H variant, the *t*-test *p*-values are shown.) **(C)** Relative abundances and standard errors associated with the undecorated complex type glycans. **(D)** Relative abundances and standard errors associated with the sialylated and fucosylated complex type glycans.

To further investigate the glycosylation differences between the wild-type TREM2 and the R47H variant, the N-glycans released from these proteins (Figure 1B, a,b) were analyzed by nano-LC/MS. Figure 3A shows ECCs of the N-glycans found in wild-type TREM2 (top) and TREM2 R47H (bottom), respectively. Each peak in Figure 3A represents individual N-glycan species, color-coded according to biosynthetic class (high mannose, undecorated complex/hybrid, fucosylated complex/hybrid, sialylated complex/hybrid, or fucosylated-sialylated complex/hybrid). Approximately 100 N-glycan compound peaks with 38 distinct N-glycan compositions were identified in wild-type TREM2, whereas over 110 N-glycan compound peaks with over 45 distinct N-glycan compositions were identified in the TREM2 R47H variant. Casual inspection revealed that N-glycosylation of wild-type TREM2 is different from that of TREM2 R47H in terms of complex/hybrid N-glycans which were not detectable in wild-type TREM2 at the retention time of 22–28 min (red box). To more precisely examine the glycosylation differences between wild-type and TREM2 R47H, the glycans detected for both proteins were quantified using the chromatogram peak areas. Figure 3B shows a bar graph of the relative abundances associated with each glycan type in R47H vs. wild-type TREM2. Glycans were grouped into high mannose type, undecorated complex/hybrid, fucosylated complex/hybrid, sialylated complex/hybrid, and fucosylated-sialylated complex/hybrid type. The high mannose type glycans were found in high abundance in both wild-type and TREM2 R47H, accounting for ~80% of the total glycans. Overall, the non-high mannose type glycan, which is less abundant in TREM2, was elevated in TREM2 R47H. Moreover, we confirmed using a *t*-test that the levels of undecorated complex/hybrid type glycans (*p* = 0.012) and fucosylated-sialylated complex/hybrid type glycans (*p* = 0.021) had statistically significant differences between wild-type and TREM2 R47H.

To more precisely quantify the differences between wild-type and TREM2 R47H, the relative abundances of individual glycans were compared. The profiles of the N-glycan structures found in both wild-type and TREM2 R47H are shown in Figure 3C (undecorated complex/hybrid type glycans) and Figure 3D (fucosylated-sialylated complex/hybrid type glycans). Wild-type TREM2, in turn, was readily distinguishable from TREM2 R47H by elevated levels of glycans consisting of [Hex]_3−6_[HexNAc]_3−5_[Fuc]_1−2_[NeuAc]_1._ Interestingly, all glycans are biosynthetically highly related, each differing from the next by few monosaccharides.

### Decreased solubility of TREM2 R47H

**Figure 4:**
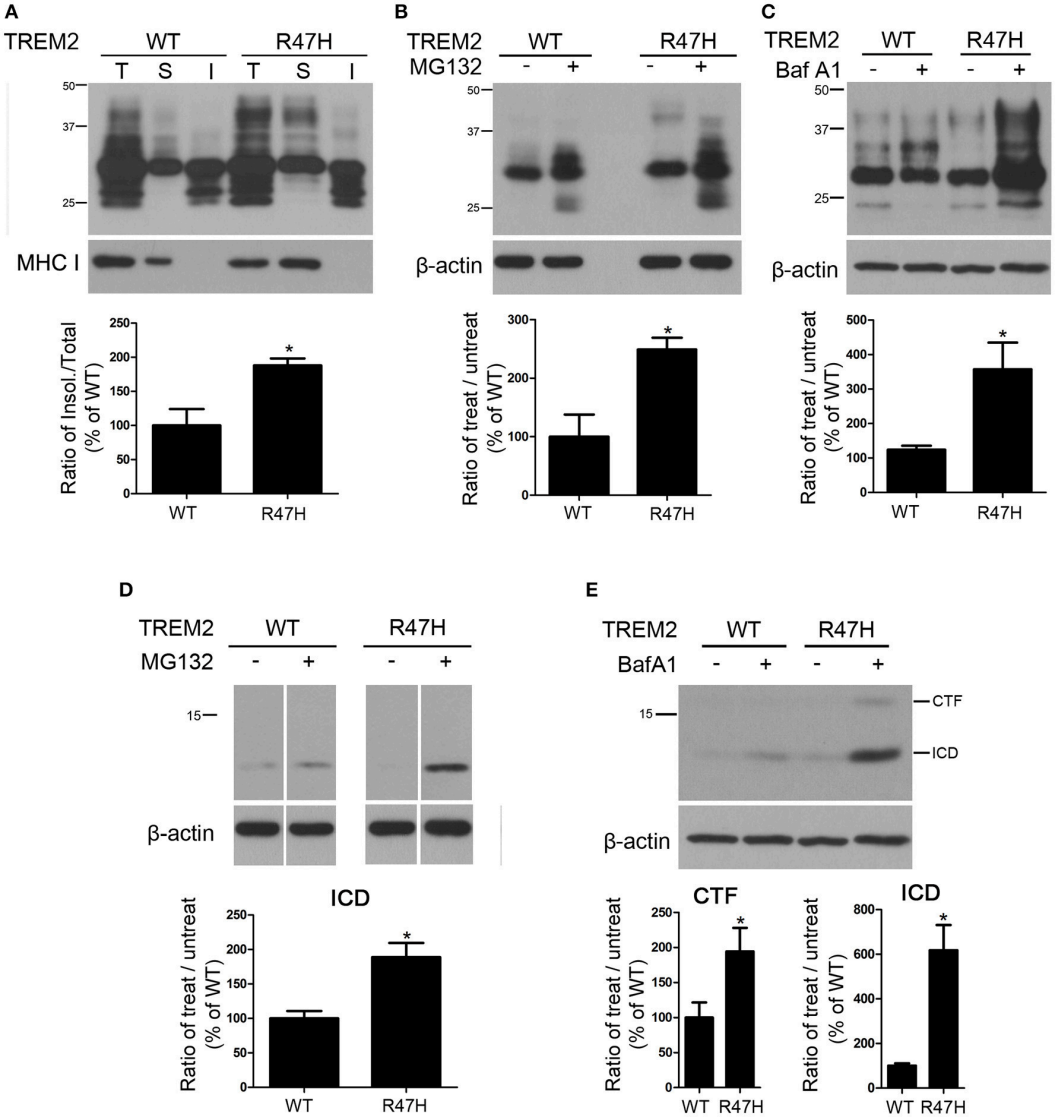
**Decreased solubility of TREM2 R47H. (A)** Protein samples from HeLa cells expressing wild-type TREM2 and its R47H variant were fractionated with detergent. TREM2 R47H was found to be increased in the detergent-insoluble fractions. Major histocompatibility complex (MHC)-I was used as a loading control for verification of fractionation. (T, total fraction; S, soluble fraction; I, insoluble fraction). **(B)** HeLa cells expressing wild-type TREM2 and its R47H variant were treated with MG 132 (10 μM) for 16 h. The TREM2 R47H levels were elevated compared to wild-type. **(C)** Following bafilomycin A1 (Baf A1) (25 nM) treatment for 16 h, TREM2 R47H expression was increased compared to wild-type. **(D,E)** The intracellular domain (ICD) bands of TREM2 are increased by treatment with MG132. Cropped images from the original blot (Supplementary Figure 2) are displayed here. Following bafilomycin A1 treatment, the CTF and ICD bands of TREM2 R47H are increased compared to wild-type. These data represent the mean ± *S.E*. from three independent experiments. ^*^P < 0.05.

To further investigate the biochemical differences between wild-type and TREM2 R47H, we tested the solubility of these proteins in triton-X100 as previously described (Kang et al., 2006). TREM2 R47H was increased in the insoluble fractions compared to the wild-type product (Figure 4A), suggesting the decreased solubility by R47H variant. We next treated cells with MG132, a proteasomal inhibitor, to block proteasomal degradation. TREM2 R47H was more elevated after MG132 treatment compared to wild-type (Figure 4B), indicating that this variant is unstable and degraded via the ER associated degradation (ERAD) pathway (Kroeger et al., 2009; D'Souza et al., 2013). We also treated cells with bafilomycin A1, a v-ATPase inhibitor, to block lysosomal degradation (Cho et al., 2014). TREM2 R47H was again more elevated after this treatment compared to wild-type (Figure 4C), again indicating that this variant is unstable and is largely degraded by lysosomes. The intracellular domain (ICD) of TREM2, which is generated after cleavage by secretases (Wunderlich et al., 2013; Kleinberger et al., 2014), was also increased in MG132- or bafilomycin A1-treated cells (Figures 4D,E).

### Increased half-life of TREM2 R47H

**Figure 5:**
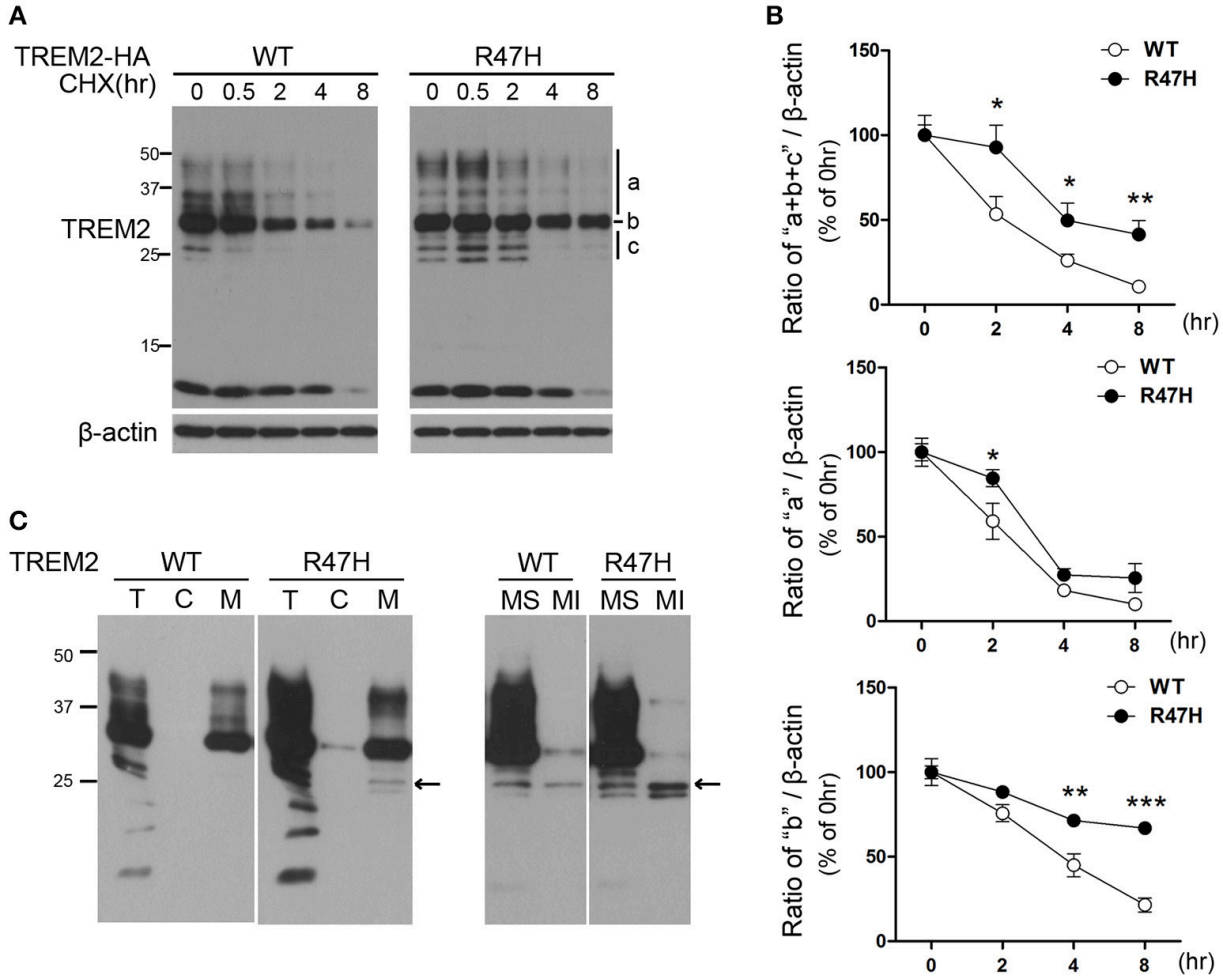
**Increased half-life of TREM2 R47H. (A)** HeLa cells transfected with wild-type TREM2 and the R47H variant were treated with cycloheximide (5 μg/μl) for the indicated times. TREM2 R47H decreased more slowly compared with wild-type. **(B)** The intensity of each band was measured using Image J and relative intensities are plotted. The data represent the mean ± *S.E*. from three independent experiments. ^*^P < 0.05, ^**^P < 0.01, ^***^P < 0.005. **(C)** Protein samples from HeLa cells expressing various types of TREM2 were fractionated with digitonin. Both wild-type and TREM2 R47H were present in the membranous organelle fraction (M). This fraction was further fractionated into membrane soluble (MS) and membrane insoluble (MI) fractions. Unglycosylated bands of TREM2 (arrow) were found only in the membranous fractions and not in the cytosolic (c) fraction.

The half-life of TREM2 was tested in transfected cells exposed to the protein synthesis inhibitor cycloheximide. In the wild-type TREM2 transfected cells, the exogenously expressed protein decreased very rapidly after cycloheximide treatment (Figures 5A,B), indicating that TREM2 is rapidly degraded under normal conditions. However, in the TREM2 R47H-transfected cells, the levels of the variant protein decreased more slowly after cycloheximide treatment compared to wild-type (Figures 5A,B), indicating a longer half-life. Interestingly, this feature was apparent in the ER forms of TREM2 R47H (Figure 5A, b and c, Figure 5B) (Park et al., 2015), but not so for the variant protein in the Golgi (Figure 5A, a, Figure 5B), suggesting that the R47H mutant has increased resistance to degradation in ER rather than to degradation after trafficking to the Golgi. Since the unglycosylated bands of TREM2 (Figure 5A, c) can reflect two forms, either in the ER or in the cytosol, we fractionated cells into cytosolic and membranous organelles, and further into soluble and insoluble membranous organelles (Figure 5C) as previously described (Kang et al., 2006). The results of this experiment indicated that the unglycosylated TREM2 form (Figure 5A, c) is in the ER membrane (Figure 5C, arrow) and not in the cytosol after retrotranslocation from the ER.

## Discussion

TREM2 mutations are associated with neurological disorders (Bird, 2013; Giraldo et al., 2013; Guerreiro et al., 2013; Luis et al., 2014), although it is not well known how TREM2 contributes to these diseases. We found in our current analyses that the AD-associated R47H variant of TREM2 has increased terminal glycosylation with complex oligosaccharides in the Golgi apparatus and decreased solubility. This may affect its ligand binding and receptor function and contribute to AD pathogenesis (Figure 7).

Since arginine 47 is located in the extracellular region of TREM2 and the R47H mutation changes the glycosylation status of the extracellular region of TREM2 (Figures 1–3), this base substitution can alter the binding of TREM2 to its ligands, its receptor function and its processing by proteases, all of which may underlie the molecular pathways by which this variant contributes to the pathogenesis of AD. Several different ligands for TREM2 have been reported previously including apolipoprotein E (Atagi et al., 2015; Bailey et al., 2015), lipids exposed upon axonal injury (Poliani et al., 2015), nucleic acids released from dying cells (Kawabori et al., 2015) and damage-associated molecular signatures found on bacteria (Daws et al., 2003; N'Diaye et al., 2009). Interestingly, the AD-associated TREM2 R47H variant shows impaired binding to apolipoprotein E or injury-associated lipids (Atagi et al., 2015; Bailey et al., 2015; Poliani et al., 2015). Hence, we speculate that the glycosylation status changes we observed herein for the R47H variant (Figures 1–3) could explain the ligand binding differences between wild-type TREM2 and TREM2 R47H (Atagi et al., 2015; Bailey et al., 2015; Poliani et al., 2015).

We and others have previously reported that the tyrosine-38 and threonine-66 residues of TREM2, which are contained within the NHD (Soragna et al., 2003), are critical for the glycosylation of this protein with complex oligosaccharides in the Golgi. This pathway is important for the surface localization of TREM2 and its processing by secretases which facilitate its normal functions (Kleinberger et al., 2014; Park et al., 2015). Y38C and T66M mutations in TREM2 produce prominent glycosylation pattern differences and impairment of trafficking to the plasma membrane (Kleinberger et al., 2014; Park et al., 2015). In our present study, we observed slight differences in the N-glycosylation of TREM2 R47H with complex oligosaccharides (Figure 1), compared with the NHD-associated Y38C and T66M variants. This difference in the glycosylation pattern seems to reflect the resulting disease severity such that NHD mutations can lead to early-onset disease and that AD is typically a late-onset disease.

TREM2 R47H has a lower solubility than wild-type (Figure 4), which could be due to its altered glycosylation status (Figures 1–3) or possible structural changes which have not yet been elucidated. This reduced solubility may cause the proteasomal and lysosomal degradation systems to become more active in order to maintain the normal steady status level of TREM2 (Figures 4B,C). However, for certain conditions that overload the normal capacity of proteasomal or lysosomal degradation, the lower solubility of TREM2 may worsen the situation, resulting in the ER stress or lysosomal degradation problems seen in neurodegenerative conditions like AD. The ICD of TREM2 is generated by ADAM12 and γ-secretase-mediated cleavage after its trafficking to the plasma membrane (Wunderlich et al., 2013; Park et al., 2015). TREM2 ICD was found to be increased in both wild-type and TREM2 R47H expressing cells by MG132 treatment (Figure 4D), indicating that TREM2 ICD is normally degraded by the proteasome although the functions of TREM2 ICD are not yet known. TREM2 ICD is highly elevated in R47H-expressing cells by about 6-fold upon exposure to bafilomycin A1 (Figure 4E), which suggests that the R47H mutation may cause TREM2 to be trafficked differently than its wild-type counterpart, resulting in the increased lysosomal degradation of TREM2 ICD.

**Figure 6:**
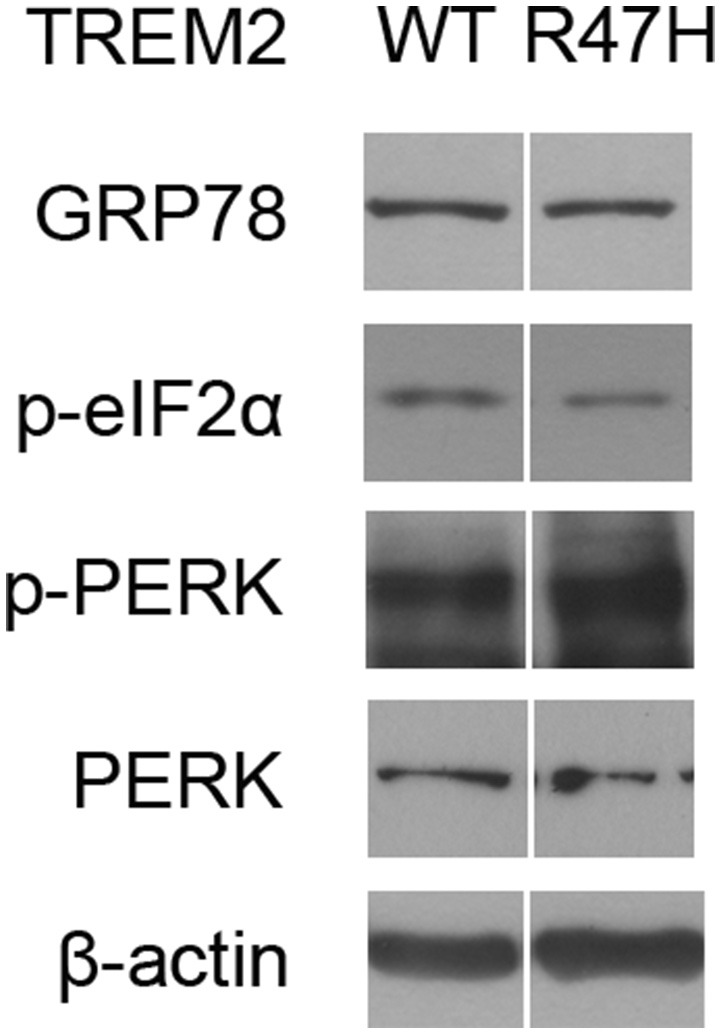
**The TREM2 R47H variant does not cause ER stress induction**. Western blots of HeLa cells transfected with wild-type TREM2 and the R47H variant indicated no significant changes in ER stress markers such as GRP78, phosphorylated eIF2α, and phosphorylated PERK. Cropped images from the original blot (Supplementary Figure 3) are displayed here.

Interestingly, the half-life of TREM2 in the ER, but not in the Golgi, is increased by the R47H mutation (Figure 5). This may be the result of an impaired retrotranslocation of TREM2 R47H from the ER due to glycosylation or structural changes. Since the unglycosylated form of TREM2 (Figure 5A, c, Figure 5B) is in the ER membrane and not in the cytosol after retrotranslocation from the ER (Figure 5C), proteasomal degradation itself seems not to be impaired by the R47H mutation. It may therefore be that the recognition and retrotranslocation of TREM2 is made much more inefficient by the R47H mutation. This however did not cause ER stress (Figure 6). The exact molecular mechanisms underlying these features of TREM2 R47H remain elusive at present and future studies will therefore be necessary.

**Figure 7:**
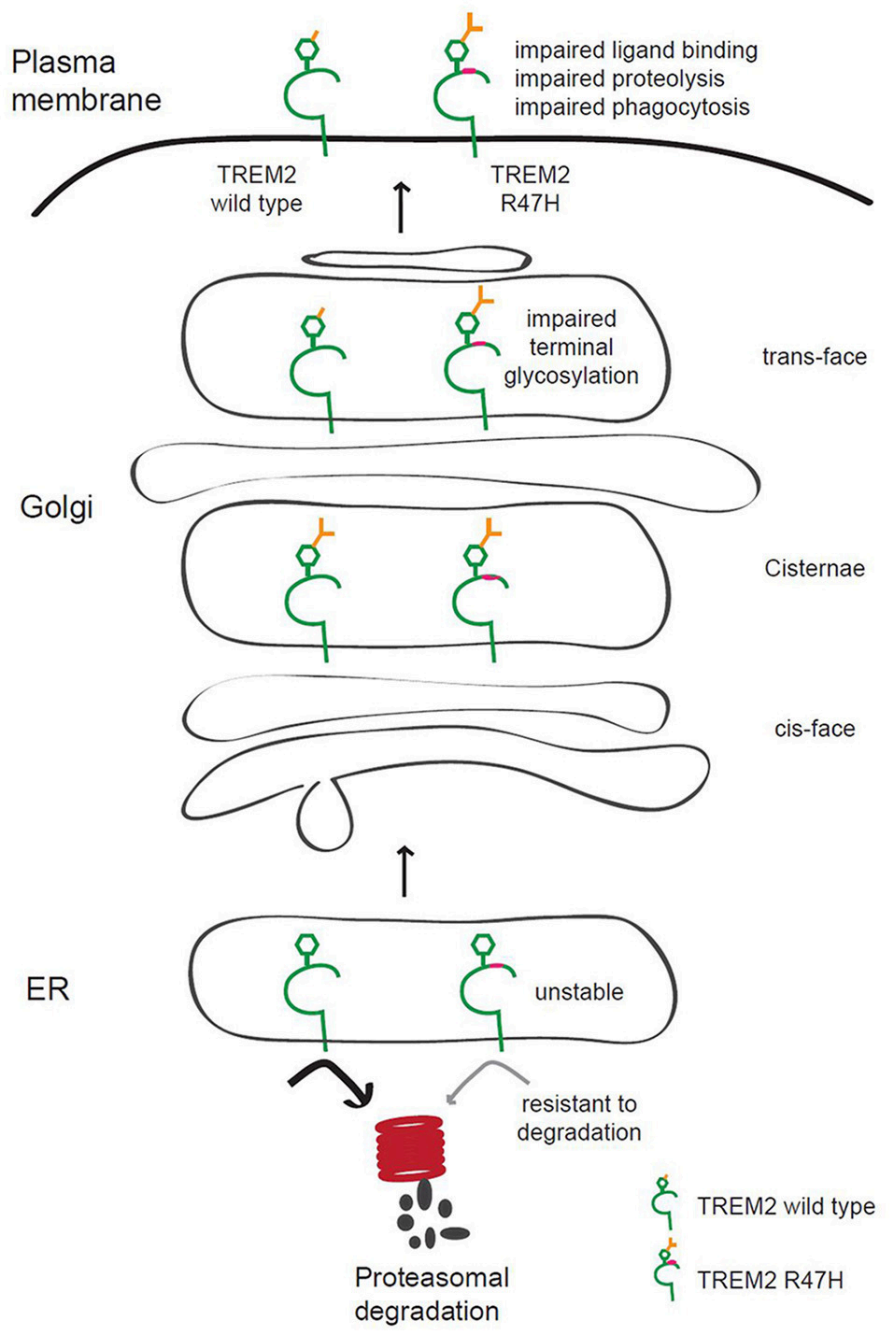
**Putative model of the role of TREM2 R47H in AD pathogenesis**. TREM2 is normally trafficked from the ER to the Golgi and plasma membrane for appropriate glycosylation in the Golgi and quality control in the ER. The AD-associated R47H variant of TREM2 shows terminal glycosylation impairment, which may disrupt ligand binding, TREM2 proteolysis or phagocytosis of microglia. TREM2 R47H is unstable and resistant to proteasomal degradation.

We demonstrate for the first time in our current study that the biochemical and molecular features of TREM2 are altered by an AD-associated R47H mutation, and that these properties of this variant protein may contribute to the pathogenesis of AD. We anticipate that the findings of our current study will be a starting point to further understand and study the biochemical characteristics of TREM2 and to possibly devise a future therapeutic strategy for AD such as normalizing the glycosylation status of TREM2 R47H.

## Author contributions

JP performed all of the experiments, analyzed the data and wrote the manuscript. IJ and HA performed and the mass spectrometry analysis and wrote this section of the manuscript. DK wrote the manuscript and supervised the experiments. SY wrote the manuscript, designed and supervised the overall experiments. All of the authors read and approved the final manuscript.

### Conflict of interest statement

The authors declare that the research was conducted in the absence of any commercial or financial relationships that could be construed as a potential conflict of interest.
