# Supplementary material for: The Alzheimer's Disease-Associated R47H Variant of TREM2 Has an Altered Glycosylation Pattern and Protein Stability
Source: Front Neurosci. 2017 Jan 18;10:618. doi: 10.3389/fnins.2016.00618 (PMC5241589; doi:10.3389/fnins.2016.00618)
Supplement: Supplementary file 1 [file DataSheet1.DOCX]

**Supplement image legend**

**Supplementary image 1. Distribution of TREM2-WT and R47H.** Immunochemistry of TREM2 with HA shows that the expression of R47H TREM2 is not significantly different from the wild-type (WT). Scale bar = 10μm

**Supplementary image 2. Uncropped full images of Figure 4D**

**Supplementary image 3. Uncropped full images of Figure 6**
