# Supplementary figures and images for: The Alzheimer's Disease-Associated R47H Variant of TREM2 Has an Altered Glycosylation Pattern and Protein Stability
Source: Front Neurosci. 2017 Jan 18;10:618. doi: 10.3389/fnins.2016.00618 (PMC5241589; doi:10.3389/fnins.2016.00618)

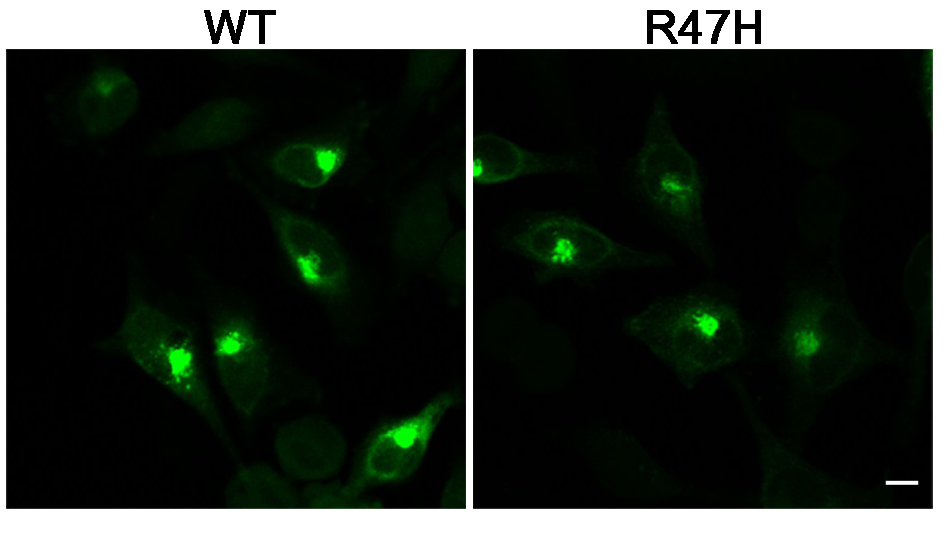

Supplement: Supplementary file 2 [file Image1.TIF]

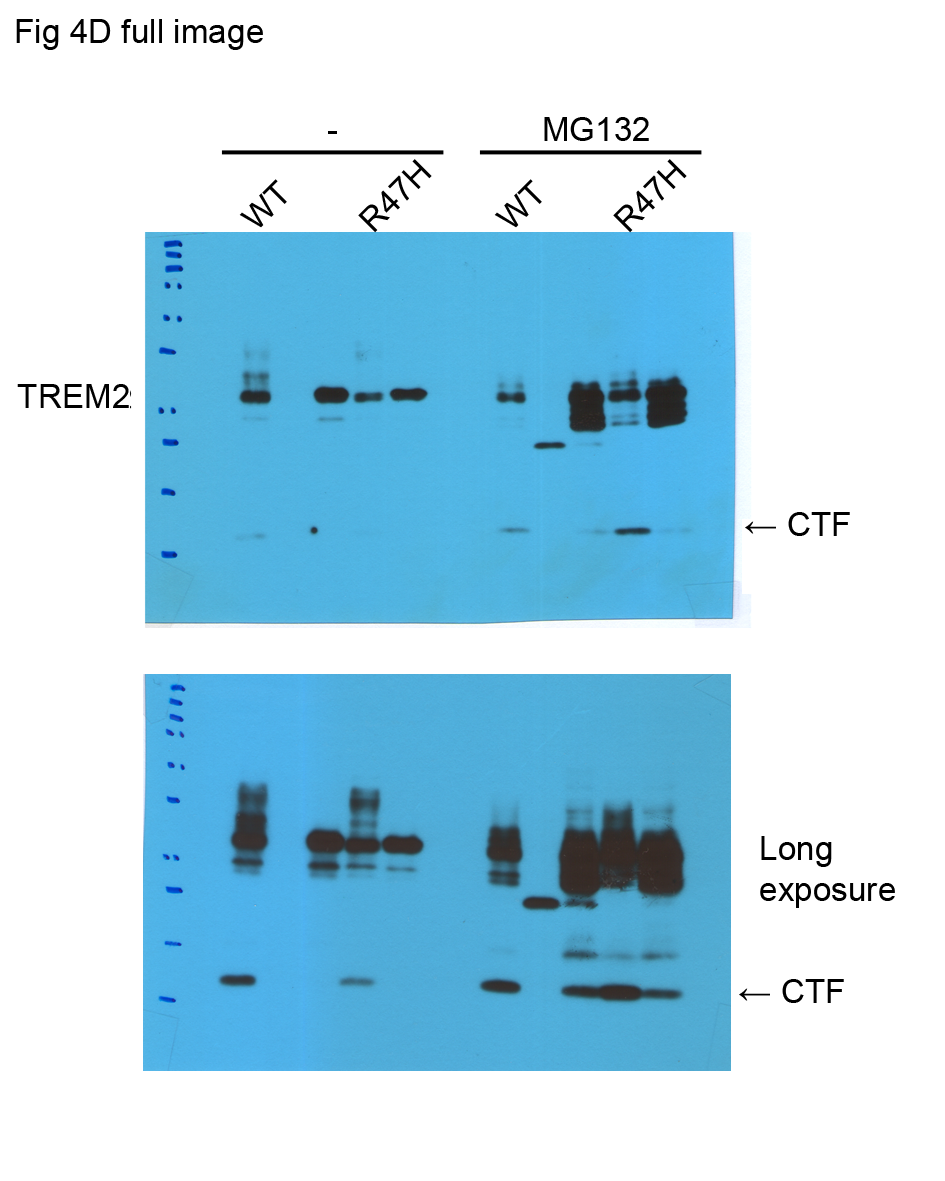

Supplement: Supplementary file 3 [file Image2.TIF]

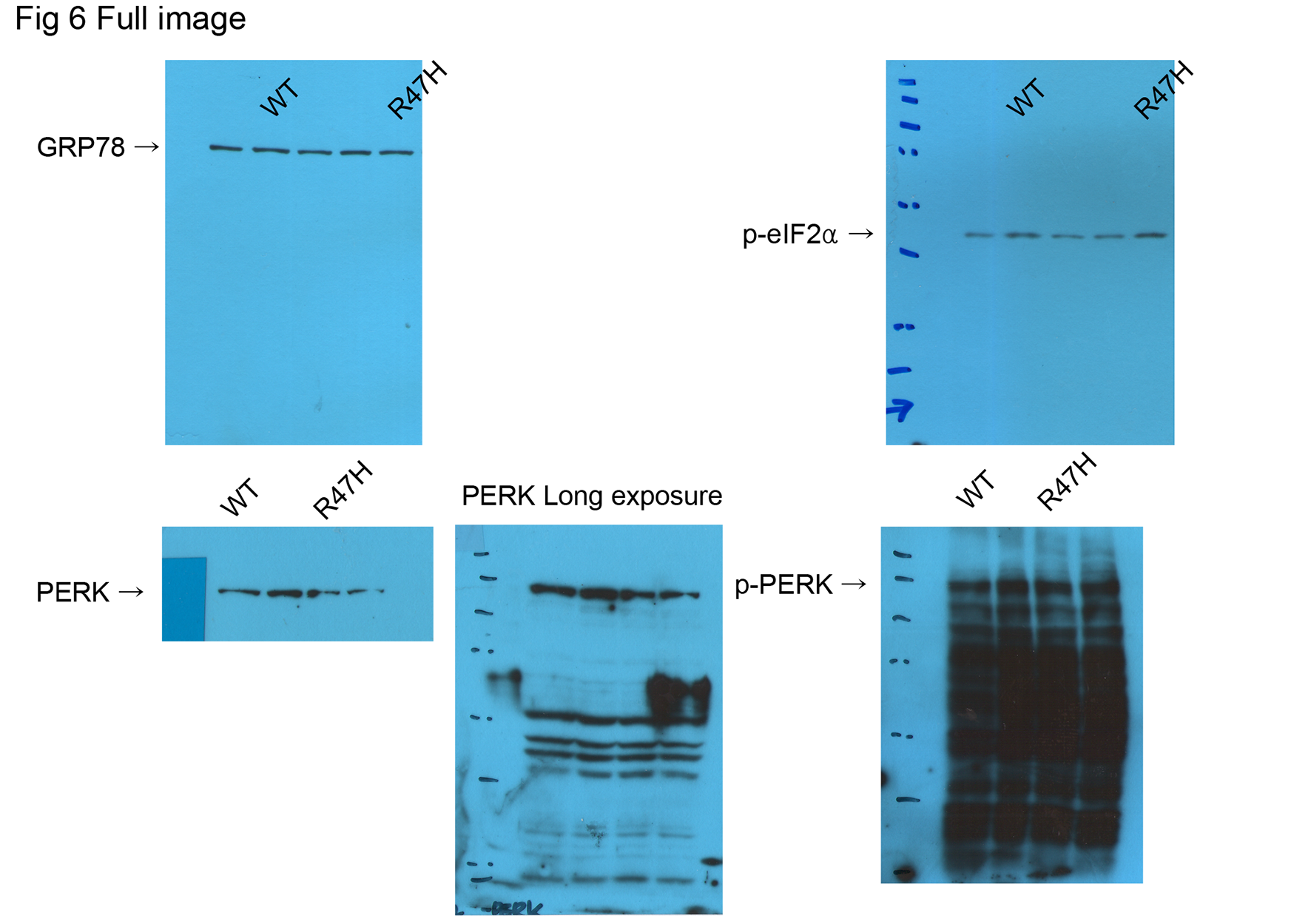

Supplement: Supplementary file 4 [file Image3.TIF]
